# CUP-Syndrom – Diagnostik aus Sicht der Pathologie

**DOI:** 10.1007/s00117-023-01143-6

**Published:** 2023-04-20

**Authors:** Chantal Pauli

**Affiliations:** Institut für Pathologie und Molekularpathologie, Universitätsspital Zürich, Rämistrasse 100, 8091 Zürich, Schweiz

**Keywords:** Unbekannter Primärtumor, Metastasen, Immunhistochemie, Linienzuordnung, Molekulare Diagnostik, Cancer of unknown primary, Neoplasm metastasis, Immunohistochemistry, Lineage differentiation, Molecular diagnostics

## Abstract

**Problemstellung:**

Die histologische und immunhistochemische Aufarbeitung von Tumormaterial aus Metastasen eines bis dahin unbekannten Primärtumors („cancer of unknow primary“, CUP) ist ein wichtiges Werkzeug zur Identifizierung ihrer Herkunft, reicht aber hierfür ohne klinisch-onkologische und radiologische Beurteilung oft nicht aus.

**Vorgehen:**

Bei der initialen CUP-Situation tragen die histologische und immunhistochemische Aufarbeitung sowie die klinisch-radiologische Korrelation wesentlich zur Identifikation des Primärtumors bei. Mittlerweile gibt es akzeptierte Richtlinien, denen man während der CUP-Diagnostik folgen kann. Mittels molekularer Diagnostik werden Veränderungen auf der Ebene von Nukleinsäuren untersucht, was u. a. auch Hinweise für den Primärtumor geben kann. Gelingt es trotz breiter und interdisziplinärer Diagnostik nicht, den Primärtumor zu identifizieren, handelt es sich um ein CUP-Syndrom. Liegt eine echte CUP-Situation vor, gilt es, den Tumor so gut wie möglich einer Tumorklasse oder einer bestimmten therapiesensitiven Untergruppe zuzuordnen, so dass die bestmögliche Therapie erfolgen kann. Für eine endgültige Zuordnung zu einem Primärtumor oder eine Einstufung als CUP ist jedoch ein Abgleich mit medizinisch-onkologischen und bildgebenden Befunden unentbehrlich.

**Schlussfolgerung:**

Beim Verdacht auf ein CUP-Syndrom ist eine enge interdisziplinäre Abstimmung zwischen Pathologie, medizinischer Onkologie und Bildgebung unerlässlich, um eine tragfähige Einstufung als CUP oder eine Identifizierung eines anzunehmenden Primärtumors zu erreichen, im Interesse einer möglichst spezifischen und wirksamen Therapie für die betroffenen Personen.

## Abstract (en)

**Problem:**

Histologic and immunohistologic workup of tumor material from metastases of a previously unknown primary tumor is important for identifying their origin, but is often insufficient for this purpose without clinical oncologic and radiologic evaluation.

**Procedure:**

In the initial cancer of unknown primary (CUP) situation, histologic and immunohistochemical workup together with clinicoradiologic correlations contribute significantly to the identification of the primary tumor. There are now accepted guidelines to follow when there is an initial CUP situation. Molecular diagnostic tools can be used to investigate changes at the nucleic acid level, which can provide clues about the primary tumor, including potential targets for therapy. If, despite broad and interdisciplinary diagnostics, it is not possible to identify the primary tumor, the diagnosis is CUP syndrome. If a true CUP situation is present, it is important to assign the tumor to a tumor class or a specific therapy-sensitive subgroup as best as possible so that the best possible treatment can be given. However, for a final assignment to a primary tumor or a final classification as CUP, a comparison with medical oncological and imaging findings is indispensable.

**Conclusion:**

When CUP is suspected, close interdisciplinary collaboration between pathology, medical oncology, and imaging is essential to achieve a viable classification as CUP or identification of a presumptive primary tumor, in the interest of providing the most specific and effective therapy for affected individuals.

Die Tumordiagnostik gehört in der Pathologie zu einer Routineaufgabe und somit auch das initiale Vorliegen eines „cancer of unknow primary“ (CUP). Die am häufigsten gestellten Fragen betreffen die Dignität einer Läsion und den Ursprung eines Tumors, letzteren insbesondere in einem metastasierten Stadium. Eine initiale CUP-Situation liegt klinisch vor, wenn zum Zeitpunkt der Probengewinnung für die histo- oder zytopathologische Untersuchung einer Metastase der zugehörige Primärtumor noch unbekannt ist.

In der Pathologie handelt es sich initial um ein CUP, wenn wir Biopsien mit einem blanken Einsendezettel ohne klinisch-radiologische Informationen erhalten, obwohl diese verfügbar wären – was sich einfach vermeiden ließe. Mit einem sorgfältigen Blick ins Mikroskop und in die Patientenakte gelingt bereits oft eine grobe Zuordnung des Tumors. Beim CUP-Syndrom handelt es sich in den meisten Fällen histologisch um Adenokarzinome mit unterschiedlichen Differenzierungsgraden sowie undifferenzierte Karzinome und seltener Plattenepithelkarzinome [21]. Bei sehr gering differenzierten Tumoren muss zuerst eine korrekte Linienzuordnung erfolgen. Unter Berücksichtigung der klinisch-radiologischen Befunde und der Histomorphologie erfolgt ein weiterführender, schrittweiser Einsatz immunhistochemischer Marker, um den Primärtumor so weit wie möglich einzugrenzen. Nicht immer gelingt es, einen spezifischen Primärtumor zu benennen, aber bereits eine Einengung auf mehrere wahrscheinliche Primärtumoren erleichtert es dem Kliniker, ihn mit möglichst geringer Patienten- und Kostenbelastung aufzudecken und eine spezifische Therapie einzusetzen. Bei etwa 25 % der Patienten mit Verdacht auf CUP ist ein Malignom in der Vorgeschichte bekannt [6], das als Ursprung der aktuellen Metastasierung in Betracht gezogen werden muss. Gelingt keine abschließende Zuordnung zu einem Primärtumor, handelt es sich definitionsgemäß um ein CUP-Syndrom [19].

## Metastasierungsmuster

**Figure 1:**
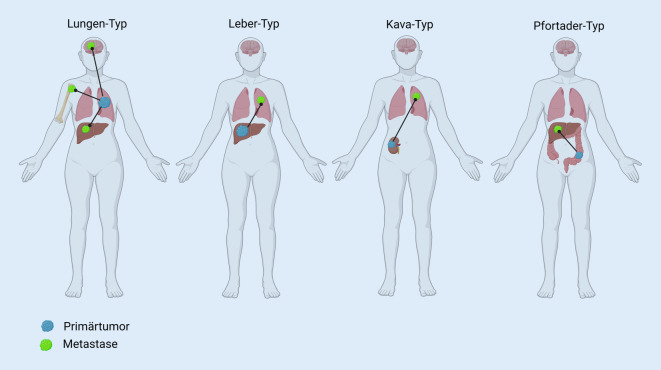

Für eine tragfähige pathologische Diagnostik ist die Kenntnis des Ortes der Biopsie unerlässlich. Bei Biopsien von Lymphknotenmetastasen geht es neben der Histomorphologie auch darum, zu erfahren, woher dieser Lymphknoten stammt. Dasselbe gilt für Biopsien aus anderen Lokalisationen. Ist es möglich, die Tumormanifestationen einem *Metastasierungstyp* zuzuordnen, kann das bereits bei der Identifikation eines möglichen Primärtumors helfen (Abb. 1). Der Ausdruck bezeichnet die Verteilung, die zu erwarten ist, wenn die Metastasierung bestimmten Pfaden folgt. Beim sog. Lungentyp finden sich u. a. Metastasen in der Leber, dem Skelett und im Gehirn, ein Muster, das man sowohl bei primären Lungentumoren findet als auch bei extrapulmonalen Primärtumoren, die bereits in die Lunge metastasiert haben und sich von dort aus weiter ausbreiten. Beim Lebertyp (ausgehend von primären Lebertumoren als auch von in die Leber metastasierten Primärtumoren außerhalb der Leber) sind die Metastasen überwiegend in der Lunge zu finden, beim Kavatyp (z. B. von der Niere ausgehend) in der Lunge, und beim Pfortadertyp findet man die Herde in der Leber, oft ausgehend vom tiefen kolorektalen Trakt [24]. Das Besondere beim individuellen CUP-Syndrom ist, dass das Metastasierungsmuster sehr heterogen sein kann und sich die häufigsten Metastasen in Lymphknoten, Leber, Abdomen, Knochen, Lunge und Gehirn finden [8].

## Pathogenese

Auch wenn sich das Verständnis von Krebsursachen und Tumorgenese durch den Fortschritt der Molekularpathologie verbessert hat, ist die genaue Pathogenese des CUP-Syndroms bislang unklar und wird hypothetisch diskutiert, wie z. B.:

- ein früh entwickelter metastatischer Phänotyp, der durch eine frühe Gefäßinvasion und eine Tendenz zur spontanen Regression oder Involution des Primärtumors gekennzeichnet ist [17];
- eine Entstehung aus versprengten embryonalen Zellen oder Geweben und eine Abstammung von adulten Stammzellen;
- auch gibt es Autoren, welche CUP als eigene Entität (primär metastasierender Tumor) mit einer äußerst hohen chromosomalen Instabilität sehen [16].

Das Fazit bleibt, dass es sich bei CUP um eine Erkrankung handelt, welche schnell metastasiert und aggressiv wächst.

## Linienzuordnung

Bei einem Tumor gilt es zunächst, die Liniendifferenzierung in epitheliale, mesenchymale, hämatologische oder melanozytäre Neoplasien einzuordnen. Die entsprechenden Hauptklassen sind Karzinome, Sarkome, Lymphome und Melanome. Beim CUP muss natürlich auch an seltenere Tumoren, wie z. B. Keimzelltumoren, Mesotheliome und auch an Paragangliome/Phäochromozytome, gedacht werden. Während bei gut differenzierten Tumoren die Linienzuordnung oft bereits histomorphologisch anhand der Hämatoxylin-Eosin(HE)-Färbung erfolgt, kann dies bei sehr gering differenzierten Tumoren oder sehr spärlichem Biopsiematerial schwierig sein. Daher ist bei der Linienzuordnung die Immunhistochemie überaus hilfreich (Tab. 1). Für die weitere immunhistochemische Aufarbeitung und die genaue Diagnose ist zumindest eine ungefähre Linienzuordnung sehr wichtig, insbesondere wenn das Material limitiert ist und nicht beliebig viele Färbungen möglich sind. Oft erfolgt die immunhistochemische Aufarbeitung in einem mehrstufigen Verfahren. Bei sehr gering differenzierten Neoplasien ist es initial sinnvoll, ein Breitspektrum-Keratin für Karzinome, CD45 für hämatologische Neoplasien und Sox10 für melanozytäre Neoplasien einzusetzen. Auch wenn die Identifizierung des Primärtumors bei einer wenig differenzierten Neoplasie im Verlauf nicht gelingen sollte, ist zumindest die korrekte Linienzuordnung (z. B. Karzinom vs. Melanom oder Sarkom) für die weitere Therapie von großer Bedeutung. Bei undifferenzierten Neoplasien kann die korrekte Linienzuordnung u. a. durch eine sog. aberrante Antigenexpression erschwert sein. Im Allgemeinen exprimieren Karzinome sog. (Zyto‑)Keratine, während mesenchymale Tumoren, beispielsweise Sarkome, Marker wie Vimentin exprimieren. Allerdings können auch Karzinome einen Verlust der Keratinexpression zeigen und häufig auch Vimentin koexprimieren. Daher wird davon abgeraten, Vimentin für eine Linienzuordnung oder in der Diagnostik mesenchymaler Neoplasien einzusetzen. Weitere Schwierigkeiten können auftreten, wenn mesenchymale Tumoren und hämatologische Neoplasien sog. Epithelmarker exprimieren – auch das kommt vor. Dies alles macht deutlich, wie wichtig es ist, die pathologischen Befunde mit den klinisch-radiologischen Befunden zu korrelieren.

**Table 1:** 

Linienzuordnung	Empfohlene initiale Immunhistochemie (Screening-Marker)	Weitere Immunhistochemie im Verlauf	Anmerkungen
Epitheliale Neoplasie – Karzinom	Breitspektrum-Keratin: AE1/AE3, OSCAR	Ergänzung mit CAM 5.2, EMA, EpCAM (Ber-EP4, MOC31)	Tab. 2 für weitere Subtypisierung
Neuroektodermale Neoplasie – Melanom	Sox 10 > S100	S100, Melan A, HMB45, CD271 (p76), PRAME, Tyrosinase	Eine frühzeitige molekulare Analyse zur Diagnosesicherung bei differenzialdiagnostisch schwierigen Fällen und für die Therapie ist empfohlen
Hämatologische Neoplasie – Lymphom	CD45	CD3, CD20, CD79a, CD30 plus subtypische Aufarbeitung	Konsultation Hämatopathologie. Einzelne Entitäten färben sich negativ für CD45: Myelome, lymphoblastische Lymphome, anaplastische Großzelllymphome und myeloide sowie Follikulär dendritische Zellsarkome
Mesenchymale Neoplasie – Sarkom	AE1/AE3, CD34, Sox10, SMA, Desmin	Je nach initialem Screening und Morphologie Marker anpassen: Myogen, Fusionssarkome	Bei Verdacht auf Fusionssarkomen molekular testen, idealerweise RNA basierter Test. Sarkome können positiv für Keratine färben. Konsultation Weichteilpathologie
Keimzelltumoren	SALL 4 > PLAP	Seminome: OCT3/4, KIT, D2-40 Embryonales Karzinom: OCT3/4, CD30 Dottersacktumor: AFP, Glypican‑3 Throphoblastischer Tumor: β‑HCG, GATA‑3, Inhibin, PD-L1	–
Mesotheliale Neoplasie – Mesotheliom	Calretinin	WT1, DS-40, K5/6, Verlust von BAP1	Bei Tumorbefall von Pleura, Perikard und Peritoneum ein Mesotheliom ausschließen. Spindelzellige Mesotheliome können die Positivität für Calretinin verlieren
Neuroendokrine Neoplasien	Synaptophysin, INSM1	Ki67 zur Einteilung von neuroendokrinen Tumoren (NET G1-G3)	Bei neuroendokrinen Tumoren ohne Primarius können CDX2 und ISLET1 helfen betreffend den Ursprung: Gastrointestinaltrakt bzw. Bauchspeicheldrüse

*AFP* α-fetoprotein, *BAP1* BRCA1 associated protein 1, *CD* cluster of differentiation, *EMA* epithelial membrane antigen, *EpCAM* Epithelial cell adhesion molecule, *EWSR1* EWS RNA binding protein 1, *hCG* human chorionic gonadotropin, *HMB45* human melanoma black 45, *OCT* octamer binding transcription factor, *PD-L1* programmed death-ligand 1, *PLAP* placental alkaline phosphatase, *SALL4* sal-like protein 4, *WT1* Wilms tumour 1

## Tumorgruppen und immunhistochemische Marker

Inzwischen gibt es zahlreiche Antikörper gegen Differenzierungs- und Zelltyp-assoziierte Tumorzellantigene mit unterschiedlicher Sensitivität und Spezifität. Die Färbungen für ein Antigen hängen von unterschiedlichen Faktoren ab, wie z. B. dem Antikörperklon, dem Färbeprotokoll, aber auch dem Zustand des zu untersuchenden Gewebes. Schlechte Fixierung und Nekrose in einem Gewebe können u. a. zu falsch-positiver Keratinexpression führen. Die aberrante Expression immunhistochemischer Marker erschwert die Diagnostik. Da es somit keinen *perfekten* immunhistochemischen Marker gibt, wird in den meisten Fällen eine Kombination unterschiedlicher immunhistochemischer Färbungen eingesetzt. Erneut ist zu betonen, dass ein pathologischer Befund immer in Zusammenschau mit der klinischen Präsentation und dem radiologischen Befund interpretiert werden muss.

### Epitheliale Neoplasien – Karzinome

Keratine sind Proteine, die an der interzellulären Filamentbildung beteiligt sind und als Marker für den epithelialen Zelltyp und dessen Linienzuordnung eingesetzt werden [12, 22]. Mehr als 50 menschliche Keratine sind bekannt, die nach einer Konsens-Nomenklatur eingeteilt werden. Der Begriff „Zytokeratine“ ist ein historischer Terminus, der immer noch weit verbreitet ist und deshalb hier auch erwähnt wird. Man unterscheidet zwei Arten von Keratinen, den sauren Typ I (K 9-23) und den neutral-basischen Typ II (K 1-8), die Heterodimere miteinander bilden. Die keratin- oder epithelspezifische Expression bleibt in den entsprechenden Karzinomen wie auch in deren Metastasen meist aufrechterhalten. Dadurch resultieren in verschiedenen Karzinomtypen charakteristische Muster, die wir uns bei der Karzinomtypisierung zunutze machen. Für die Linienzuordnung bei wenig differenzierten Neoplasien bieten sich sog. Pan-Keratinmarker wie OSCAR oder MNF-116 an, aber auch Cocktails aus zwei Antikörpern gegen saure und basische Keratine AE1/AE3. CAM5.2 (Keratin 7 und 8) werden beim Screening oft ergänzt. Für die Tumordiagnostik wichtig sind im wesentlichen K5/K6, K7 und K20, ferner noch K8/K18 [23]. In Europa weit verbreitet und entsprechend der Richtlinien der European Society for Medical Oncology (ESMO) von großer Relevanz ist insbesondere der kombinierte Einsatz von K7 und K20, woraus eine Karzinom-Phänotypen-Einteilung in K7+/K20+, K7+/K20−, K7−/K20+ und K7−/K20− resultiert ([25]; Tab. 2). K7 als sog. *duktales* Keratin wird in der Mehrzahl der Adenokarzinome stark exprimiert, es fehlt aber weitgehend in Kolorektal- und Prostatakarzinomen. K20 als sog. *intestinales* Keratin kommt charakteristischerweise in kolorektalen Adenokarzinomen vor [15]. Bei soliden Karzinomen sind K5/K6 und K8/K18 zur Erkennung des plattenepithelialen bzw. zylinderepithelialen Grundcharakters sinnvoll [23]. Das Epithelialmembran-Antigen (EMA, aka MUC1) wird oft in der Diagnostik für Karzinome, insbesondere Adenokarzinome eingesetzt, zusammen mit den Keratinen. Bei den Adenokarzinomen findet sich eine zytoplasmatische Anfärbbarkeit, bei Mesotheliomen hingegen eine starke membranäre Anfärbung. Des Weiteren findet sich eine EMA-Positivität bei mesenchymalen Tumoren wie dem Synovialsarkom, dem epithelioiden Sarkom und bei Chordomen, welche oft auch eine Positivität für Keratine zeigen. Die Kombination EMA-Positivität und Keratin-Negativität findet sich insbesondere bei Meningeomen, Perineuriomen und hämatologischen Neoplasien, wie z. B. Plasmazellneoplasien [7].

**Table 2:** 

Initiale Keratinmarker	Tumorentitäten	Zusätzliche Marker
K7+/K20−	Lunge (NSCLC [Adeno] und SCLC)	TTF1, SMARCA4, Synaptophysin
	Schilddrüse	Thyroglobulin, TTF1, Pax8
	Mamma	GATA3, Sox10, TRPS1, ER, PR, Her2
	Oberer GI-Trakt/pankreatobiliär	CDX2, K19, SMAD4, ARID1A, BAP1
	Endometrium, Endozervix, Ovar (z. B. serös)	Pax8, ER, PR, WT1, p53
	Nierenzellkarzinom (z. B. klar und papillär)	Pax8, Pax2, Racemase, CD10
	Speicheldrüse	GATA3, S100, Sox10, AR, HER2
	Blase	GATA3, p63
K7+/K20+	Blase	GATA3, p63
	Oberer GI-Trakt/pankreatobiliär	CDX2, K19, SMAD4, ARID1A, BAP1
	Rektum (Ausnahmen)	CDX2, SATB2
K7−/K20+	Kolorektal, oberer GI-Trakt (Ausnahme)	CDX2, SATB2
	Merkel-Zell-Karzinom	Synaptophysin, MCPyV
K7−/K20−	Nierenzell-Karzinom	Pax8, Pax2, Racemase, CD10
	Hepatozelluläres Karzinom	Arginase1, HepPar1
	Keimzelltumoren	SALL4, PLAP, OCT3/4, C‑Kit, Glypican‑3, Beta-HCG, CD30
	Prostata	PSMA, NKX3.1, PSA
	Magen	CDX2
	Kleinzelliges/neuroendokrines Karzinom	Synaptophysin, INSM1, Lunge: TTF1
	Adrenokortikales Karzinom	SF1, Calretinin, Inhibin, Melan‑A
	Plattenepithelkarzinome	p40, p63, K5/6

*AR* Andorgen-Rezeptor, *ARID1A* AT-rich interactive domain-containing protein 1A, *BAP1* BRCA1 associated protein 1, *CD10* cluster of differentiation 10, *CDX2* 8 Caudal type homeobox 2, *CK* cytokeratin, *ER* estrogen receptor, *GI* gastrointestinal, *HepPar1* hepatocyte specific antigen, *HER2* human epidermal growth factor receptor 2, *IHC* immunohistochemistry, *INSM1* insulinoma-associated protein 1, *NSCLC* non-small-cell lung cancer, *p* tumour protein, *PAX2* paired box gene 2, *PAX8* paired box gene 8, *PgR* progesterone receptor, *PLAP* placental alkaline phosphatase, *PSMA* prostate-specific membrane antigen, *SALL4* sal-like protein 4, *SATB2* special AT-rich sequence-binding protein 2, *SCLC* small-cell lung cancer, *SF1* steroidogenic factor 1, *SMAD4* mothers against decapentaplegic homologue 4, *SMARCA4* SWI/SNF related, matrix associated, actin dependent regulator of chromatin, subfamily A, member 4, *SMARCB1* SWI/SNF related, matrix associated, actin dependent regulator of chromatin, subfamily B, member 1, *TTF1* thyroid transcription factor 1, *WT1* Wilms tumor 1

### Neuroektodermale Neoplasien – Melanome

Marker wie MART1 („melanoma antigen recognized by T cells 1“), MiTF („microphthalmia transcription factor“), HMB45 („human melanoma black-45“) und Tyrosinase sind in der Melanomdiagnostik weit verbreitet und werden oft auch in einer sog. Pan-Melan-Cocktail-Form angeboten. Weiter werden S100 und Sox10 („SRY-related HMG-box gene 10“) oft für Screenings im CUP-Setting eingesetzt, da Melanome bei der Metastasierung sog. spezifische und sensitive Marker verlieren können und z. B. auch spindelzellige und desmoplastische Melanome die herkömmlichen melanozytären Marker nicht exprimieren. Neuere Marker, die heute auch im metastasierten Setting und bei der Differenzialdiagnose des Melanoms zum Einsatz kommen, sind PRAME („preferentially expressed antigen in melanoma“) und CD271, ein Rezeptor in Nervenzellen, der analog auch in Melanozyten und Melanomzellen exprimiert wird [29]. Generell als Screening-Marker empfiehlt sich Sox10 und S100, wobei Sox10 gegenüber S100 vorgezogen werden sollte. Wichtig anzumerken ist, dass bei spindelzelligen Neoplasien noch unklarer Zuordnung mit S100, Sox10, PRAME und CD271 differenzialdiagnostisch auch an einen peripheren Nervenscheidentumor gedacht werden muss.

### Mesenchymale Neoplasien – Sarkome

Vimentin ist immer noch weit verbreitet in der Diagnostik mesenchymaler Neoplasien. Es handelt sich dabei jedoch um einen sehr unspezifischen Marker, welcher breit von Tumoren unterschiedlicher Linienzuordnung exprimiert wird und mit Vorsicht gewertet werden muss. Ein gewisser Stellenwert von Vimentin bleibt lediglich bei der Unterscheidung Endometrium- vs. Zervixkarzinom sowie klarzelligem vs. chromophobem Nierenzellkarzinom und Onkozytom [14, 30]. Es gibt folglich keinen sarkomspezifischen Marker, und bei Verdacht auf ein Sarkom muss neben der Immunhistochemie die molekulare Diagnostik mit einbezogen werden.

### Hämatologische Neoplasien – Tumoren

CD45 („aka leukocyte common antigen“, LCA oder CLA) ist ein Pan-Leukozytenmarker, der in der Regel alle hämatologischen Neoplasien färbt, abgesehen von einigen Myelomen und lymphoblastischen Lymphomen, anaplastischen Großzelllymphomen und myeloiden sowie follikulär dendritischen Zellsarkomen.

### Neuroendokrine Tumoren

Bei Triple-Negativität für Pan-Keratin/CD45/Sox10 oder S100 sollte neben einem Sarkom insbesondere bei einer epithelioiden Zytomorphologie auch an ein Paraganglion/Phäochromozytom gedacht werden. Bei Tumoren mit regelmäßigem soliden, trabekulären bis gyriformen Drüsenwachstumsmuster und einheitlichen Kernen mit einem *Salz-und-Pfeffer*-ähnlichen Chromatin muss eine neuroendokrine Neoplasie ausgeschlossen werden. Neben Synaptophysin bietet sich auch INSM1 („insulinoma-associated protein 1“) als guter Screening-Marker dafür an. Diese Färbungen sollten auch bei weniger differenzierten Tumoren durchgeführt werden, um ein kleinzelliges Karzinom oder ein großzelliges neuroendokrines Karzinom der Lunge nicht zu verpassen. Die Positivität für CDX2 („caudal type homeobox 2“) und ISLET1 („ISL LIM homeobox 1“) kann bei neuroendokrinen Neoplasien auf eine primäre Lokalisation im Gastrointestinaltrakt respektive in der Bauchspeicheldrüse hinweisen.

### Mesotheliale Neoplasien – Mesotheliom

Mesotheliome können aufgrund ihrer Positivität für Keratine fälschlicherweise als Karzinome interpretiert werden. Bei einer Tumormanifestation in der Pleura, im Peritoneum und im Perikard muss differenzialdiagnostisch immer ein Mesotheliom in Betracht gezogen werden. Als nützliche positive Marker zur Diagnose eines Mesothelioms sind Calretinin, WT‑1, D2‑40 und als negative Marker BerEP4 und BAP1 (Verlust) zu nennen. Sarkomatoide Mesotheliome können Keratin-positiv ausfallen und Calretinin in der Expression verlieren.

### Keimzelltumoren

SALL4 oder PLAP („placental alkaline phosphatase“) sind geeignet im Screening für Keimzelltumoren, wobei SALL4 aufgrund der besseren Sensitivität bevorzugt werden sollte. SALL4 wie auch PLAP können jedoch auch in Adenokarzinomen mit einer sog. hepatoiden Differenzierung positiv ausfallen.

## Linienspezifischere Aufarbeitung

Nachdem eine Linienzuordnung erfolgt ist, kommen Marker mit höherer *Organspezifität* zum Einsatz (Tab. 2). Eine strenge Organspezifität liegt jedoch nur sehr selten vor. Zusammen mit den ESMO-Guidelines werden in der Literatur unterschiedliche Algorithmen zur immunhistochemischen Tumorklassifikation vorgeschlagen [9, 18]. Damit kann im Idealfall eine Bestimmung des Primärtumors erreicht werden.

### Lungenkarzinome

Bei K7-Positivität und K20-Negativität sowie einer pulmonalen Raumforderung muss an einen Primärtumor der Lunge gedacht werden. Etwa 60–80 % der schlecht differenzierten und metastasierten Lungenadenokarzinome färben sich positiv für den Schilddrüsen-Transkriptionsfaktor 1 (TTF1). Bei K7-Positivität und TTF1-Negativität, aber hochgradigem klinischem und radiologischem Verdacht auf einen Primärtumor der Lunge bietet sich zudem eine SWI/SNF-Komplex-bezogene Färbung für SMACA4 an. TTF1-negative Lungenadenokarzinome zeigen oft einen Verlust der SMARCA4-Kernfärbung, und diese Konstellation kann die Diagnose eines Primärtumors der Lunge unterstützen [31]. Napsin A kann in diesem Zusammenhang in einem Panel zusammen mit TTF1 eingesetzt werden, hat bei TTF1-Negativität für die Lungenkarzinomdiagnostik aber keinen nennenswerten Stellenwert. Bei isolierter K7-Positivität muss anhand der radiologischen Morphologie entschieden werden, ob eine pulmonale Raumforderung einer Metastase entspricht, oder ob allfällige mediastinale und hiläre Lymphknoten vorliegen, und von einem primären Tumor der Lunge ausgegangen werden kann. Im Übrigen hat sich TTF1 zusammen mit Thyreoglobulin ebenfalls sehr gut für die Schilddrüsendiagnostik bewährt [3].

### Gastrointestinale Tumoren

Bei Adenokarzinomen des oberen Gastrointestinaltrakts ist die K7-Färbung in den meisten Fällen positiv bei Negativität für K20. Für kolorektale Adenokarzinome ist neben dem charakteristischen Phänotyp K7−/K20+ der Transkriptionsfaktor CDX2 von diagnostischer Bedeutung. Mindestens 80 % der kolorektalen Karzinome zeigen den klassischen K7-negativen, K20- und CDX2-positiven Immunphänotyp. K20 und die CDX2-Färbung sind normalerweise diffus und stark gefärbt, können jedoch bei Mikrosatelliten-instabilen Tumoren auch verlorengehen. Gelegentlich zeigen Karzinome des oberen GI-Trakts, insbesondere des Magens, und seltener auch pankreatikobiliäre Adenokarzinome einen kolorektalen Immunphänotyp. In dieser Konstellation kann das spezielle AT-reiche sequenzbindende Protein 2 (SATB2) eingesetzt werden. Dieser nukleäre Marker ist in Kombination mit K7 und K20 ein spezifischer Marker für kolorektale Tumoren. Bei Adenokarzinomen mit K7-Negativität und K20- sowie CDX2-Positivität und fehlendem Primärtumor im unteren GI-Trakt geht man von einem „colon-like CUP“ aus und behandelt analog.

Bei multiplen Läsionen in der Leber muss zwischen einem metastasierten Primärtumor innerhalb der Leber und Metastasen eines extrahepatischen Primärtumors unterschieden werden. Die Radiologie hat in der Diagnostik des hepatozellulären Karzinoms Vorrang, kann aber histomorphologisch und immunhistochemisch mit Markern wie Arginase1 und Hepar1 unterstützt werden. Die Diagnose intrahepatischer Cholangiokarzinome durch die Immunhistochemie bleibt aufgrund des Fehlens spezifischer Marker weiterhin eine Ausschlussdiagnose. Beim Cholangiokarzinom handelt es sich in den meisten Fällen um ein K7-positives Karzinom, welches keinem anderen Primärtumor zugeordnet werden kann. Immunhistochemisch kann ein Verlust von BRCA1-assoziiertem Protein 1 (BAP1) oder des AT-reichen interaktiven Domäne enthaltenden Proteins 1A (ARID1A) die Diagnose eines intrahepatischen Cholangiokarzinoms unterstützen. Die endgültige Diagnose kann jedoch nur nach Korrelation mit der klinischen und insbesondere radiologischen Präsentation gemacht werden [18]. Gleiches gilt für Pankreaskarzinome, bei denen SMAD4 (DPC4, MADH4) hinzugezogen werden kann. Da SMAD4 insbesondere auch bei anderen GI-Tumoren immunhistochemisch verlorengehen kann, muss die abschließende Diagnose mittels Radiologie und Klinik erfolgen.

### Urogenitale Tumoren

Bei osteoplastischen Knochenmetastasen beim Mann muss immer ein Prostatakarzinom ausgeschlossen werden. Die Immunhistochemie für das klassische prostataspezifische Antigen (PSA) ist weitverbreitet, färbt sich aber gelegentlich auch in anderen Tumoren positiv und kann andererseits bei geringer Differenzierung im metastasierten Stadium verlorengehen. Als Screening-Marker bietet sich PSMA (prostataspezifisches Membranantigen) und NKX3.1 („NK3 homebox 1“) an, da die Expression dieser Marker besser erhalten bleibt [10].

Uroplakin II–III sind sehr spezifische Marker für Urothelkarzinome und deren Metastasen. Sie sind jedoch nicht sehr sensitiv, was in einer diagnostischen Aufarbeitung bei CUP berücksichtigt werden muss. Eine besondere Herausforderung stellt das Nierenzellkarzinom-ähnliche CUP-Syndrom dar, insbesondere aufgrund der Therapie. Es gibt Patienten und Patientinnen mit metastasierter Erkrankung, welche histomorphologisch und immunhistochemisch mit einem Nierenzellkarzinom kompatibel sind, jedoch ohne Läsion in der Niere. Aktuelle Literatur zeigt, dass diese Patienten und Patientinnen von einer nierenspezifischen Therapie mit Tyrosinkinase- oder Checkpoint-Inhibitoren profitieren können. Wichtig ist hier ein breites Marker-Panel, welches diese Diagnose unterstützt, wie PAX8, PAX2, CD10 und Racemase. Der Marker RCC („renal cell carcinoma“) sollte nur im Zusammenhang mit anderen Markern angewendet werden.

### Mammakarzinom und gynäkologische Tumoren

Bei der Frau muss beim Vorliegen von Metastasen immer ein Mammakarzinom als Primärtumor ausgeschlossen werden. Immunhistochemisch bietet sich GATA3 als bislang sensitivster Screening-Marker für Mammakarzinome an. Rund 100 % der Östrogen-positiven Mammakarzinome sind GATA3-positiv. Bei Östrogenrezeptor-negativen Brustkarzinomen ist GATA3 in 70–80 % der Fälle positiv. Auf GCDFP-15/BRST2 oder Mammaglobin kann bei fehlendem Mehrwert verzichtet werden. Bei triple-negativem Mammakarzinom sollte Sox10 oder TRPS1 („trichorhinophalangeal syndrome type 1“) als Screening-Marker eingesetzt werden [2].

Bei Frauen mit einer peritonealen Aussaat muss an einen Primärtumor des Ovars, der Tuben, des Uterus oder an ein primäres peritoneales Karzinom gedacht werden. Pax8 als sog. Pan-Müllerischer Marker (Ovar, Endometrium, Endozervikal) wird oft zusammen mit WT1 und p53 (seröse Ovarialkarzinome) bei der Suche nach einem gynäkologischen Primärtumor eingesetzt. Beim Vorliegen einer isolierten Peritonealkarzinose durch ein seröses Adenokarzinom ohne sicher nachweisbaren Primärtumor liegt ein sog. „ovar-like CUP“ vor, und die Therapie sollte analog eines high-grade serösen Ovarkarzinoms erfolgen [5].

## Häufigste Fehlinterpretationen

**Figure 2:**
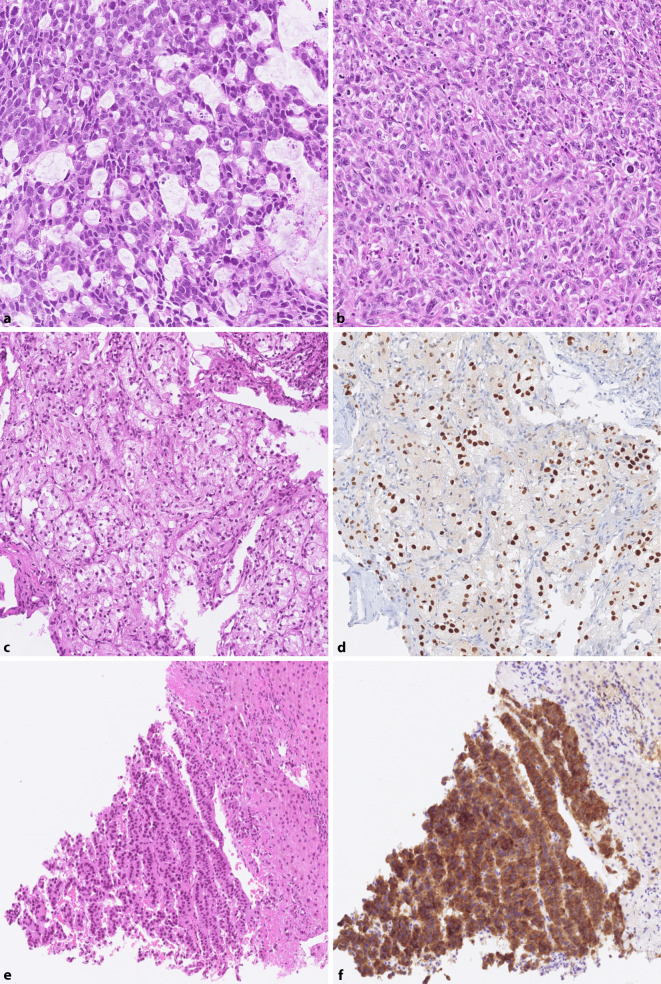

Die Erfahrung in der Referenzpathologie (Institut für Pathologie und Molekularpathologie, Universitätsspital Zürich, Schweiz) für die CUPISCO-Studie, in welcher der Screening-Prozess einen histopathologischen Review mit klinisch-radiologischer Korrelation beinhaltete, ging mit einer hohen Fehlerrate im initialen Screening einher [25], der nur in engem Austausch zwischen den Fachdisziplinen Pathologie, medizinische Onkologie und Radiologie begegnet werden konnte. Fälschlich als CUP eingestuft wurden initial am häufigsten Karzinome der Lunge, der Mamma, der Leber (hier insbesondere das intrahepatische cholangiozelluläre Karzinom), der Bauchspeicheldrüse und der Niere. Fehlinterpretationen bei der rein pathologischen Aufarbeitung zeigten sich am häufigsten bei neuroendokrinen Tumoren, Sarkomen und Mesotheliomen, welche als wenig differenzierte Karzinome diagnostiziert wurden (Abb. 2).

## Molekulare Diagnostik

Bei unklarem Primärtumor ist eine molekulare Diagnostik zur genaueren Analyse von Nukleinsäureveränderungen empfohlen, da diese teilweise auf den Primärtumor schließen lassen. Dies ist auch von besonderer Relevanz, da molekulare Veränderungen u. a. die potenzielle Behandlung mit zielgerichteten Therapien oder Immuncheckpoint-Inhibitoren ermöglichen. Es bieten sich im Setting eines CUP-Syndroms sog. Pan-Krebs-Panels an, welche relevante molekulare Alterationen über verschiedene Entitäten hinweg abdecken und dazu den Mikrosatellitenstatus sowie die Tumormutationslast mitmessen. In Einzelfällen kann das molekulare Profil mehr Klarheit schaffen oder Hinweise in Bezug auf den vermeintlichen Primärtumor geben. Als Beispiel wäre hier u. a. Transmembran-Serinprotease 2 (TMPRSS2) in Prostatakarzinomen oder anaplastische Lymphomkinase (ALK) und ROS-Proto-Onkogen 1 (ROS1) in nichtkleinzelligen Lungenkarzinomen (NSCLC) zu nennen. Eine Liste von genomischen Aberrationen, welche die Diagnose spezifischer Neoplasien unterstützen, findet sich in Tab. 3 [18]. In mehreren retrospektiven Studien konnte gezeigt werden, dass bei auf Gewebe- oder Flüssigbiopsie basierten zellfreien zirkulierenden Tumor-DNA(cfDNA)-Analysen in 65–90 % der Patienten mit CUP ≥ 1 onkogene Treibermutation identifiziert werden kann [26]. Die häufigsten molekularen Veränderungen bei Patienten mit CUP finden sich in den Genen *TP53* (37–55 % der Fälle), gefolgt von *KRAS* (18–20 %), *PIK3CA* (9–15,4 %), *ARID1A* (~ 11 %) und *EGFR* (~ 6–17 %) [28]. Viele dieser Genveränderungen gelten als schwierig zu therapieren, andere hingegen wie *EGFR* bei Lungenkrebs und *PIK3CA* bei Brustkrebs sind mittlerweile zielgerichtet behandelbar. Die meisten Daten zur therapeutischen Anpassung an genomische Veränderungen bei CUP stammen jedoch aus Fallberichten [26]. In den letzten Jahren konnte gezeigt werden, dass mittels Genexpressionsanalyse basierend auf Ribonukleinsäure (RNA) in rund 70–80 % der Fälle eine Zuordnung eines Primärtumors möglich ist [27]. Mittels miRNA-Profiling gelang es in rund 70 %, den Ursprung von Tumoren zu identifizieren [11]. Weiter kann die Erstellung von DNA(Desoxyribonukleinsäure)-Methylierungs- und Kopienzahlvariation(CNV)-Profilen das Potenzial haben, den Ursprung von Tumoren zu entlarven, die als Krebs mit unbekanntem Primärtumor klassifiziert wurden. Ein von Forschern entwickelter diagnostischer Assay, EPICUP, sagte in 87 % von 216 Fällen die Lokalisation des primären Tumors voraus. Diese Vorhersagen wurden durch verschiedene Tests, einschließlich Immunhistochemie, verifiziert [20]. Mit dem Einsatz von künstlicher Intelligenz (KI) und maschinellem Lernen auf DNA- und RNA-Sequenzierungsdaten wurde kürzlich gezeigt, dass in rund 71,7 % von CUP-Fällen eine akkurate Vorhersage über den Primärtumor gemacht werden konnte, woraufhin in 41,3 % der Fälle die Diagnose von Pathologen und Pathologinnen angepasst wurde [1]. Die Potenz dieser molekularbiologischen Analysen ist enorm und kann komplementär eingesetzt werden. Ob solche neueren, methodisch doch aufwendigen und teuren Technologien den Einzug in die klinisch-pathologische Routinediagnostik finden, wird sich zeigen. Problematisch bleibt, dass diese z. B. genexpressionsbasierte sog. „site-specific“ eingesetzten Therapien, bislang keinen therapeutischen Benefit gegenüber der standardisierten empirischen Chemotherapie mit Carboplatin-Paclitaxle oder Cisplatin-Gemcitabine gezeigt haben [13]. Ob eine maßgeschneiderte Behandlung auf der Grundlage von genomischem Profiling im Vergleich zum Standard-Chemotherapie-Ansatz für Patienten mit CUP vorteilhaft ist, wird sich in der prospektiven, randomisierte Phase-2-Studie CUPISCO (NCT03498521) zeigen, deren Rekrutierung kürzlich abgeschlossen wurde. Hier wird aktuell untersucht, ob sich mehrere Klassen zielgerichteter Therapien (einschließlich Immuntherapeutika und Inhibitoren der Rezeptor-Tyrosinkinasen) vorteilhaft im Vergleich zum Standard-Chemotherapie-Ansatz für Patienten mit CUP auswirken.

**Table 3:** 

Tumorentität	Genomische Alteration	Histologie
Nichtkleinzelliges Lungenkarzinom	*ALK- *oder* ROS1*-Fusionen	–
Intrahepatisches cholangiozelluläres Karzinom	*FGFR2*-Fusionen	–
Neoplasien der Speicheldrüsen	*ETV6::NTRK3* *MYB* Fusionen *MYBL2*-Fusionen *EWSR1::ATF1* *MAML2*-Fusionen *PLAG1* oder *HMG2* Fusionen *RKD1*-Mutationen	Sekretorisches Karzinom Adenoidzystisches Karzinom Adenoidzystisches Karzinom Hyalinisierendes klarzelliges Karzinom Mukoepidermoides Karzinom Pleomorphes Adenom mit Ex-PA-Karzinom Polymorphes Adenokarzinom
NUT-Karzinom	*NUTM1*-Fusionen	–
Prostatakarzinom	*TMPRSS2::ERG*	–
Sarkome und andere	*EWSR1::FLI1/ERG* *EWSR1::WT1* *ETV6::NTRK3* *EWSR1::POU5F1* *TFE3*-Fusionen *NAB2::STAT6* *NR4A3*-Fusionen *SMARCB1*-Mutationen *SS18(SYT)*-Fusionen *COL1A1::PDGFB* *KIT*-Mutationen *FUS-CREB3L2/CREB3L1* *DDIT3*-Fusionen *HEY1::NCOA2* *BCOR*-Fusion und Mutationen	Ewing-Sarkom Desmoplastischer Rundzelltumor Infantiles Fibrosarkom Myoepitheliom/myoepitheliales Karzinom Alveoläres Weichteilsarkom, EHE, PEComa Solitärer fibröser Tumor Extraskeletales myxoides Chondrosarkom Maligner rhabdoider Tumor/epitheloides Sarkom Synovialsarkom Dermatofibrosarkom GIST Low grade fibromyxoides Sarkom Sklerosierendes epitheloides Fibrosarkom Myxoides und rundzelliges Liposarkom Mesenchymales Chondrosarkom Rund- bis Spindelzellsarkome
HCC	*PRKACA*-Fusionen	Fibrolamelläres hepatozelluläres Karzinom
RCC	*TFE3-*Fusionen *TFEB*-Fusionen *VHL*-Mutationen	Translokationsassoziiertes Nierenzellkarzinom Klarzelliges Nierenzellkarzinom
Mamma	*ETV3*-Fusionen	Sekretorisches Karzinom

*ALK* anaplastic lymphoma kinase, *ATF1* activating transcription factor 1, *BCOR* BCL6 corepressor, *CCA* cholangiocarcinoma, *COL1A1* collagen, type 1, alpha 1, *CREB3L1* CAMP responsive element binding protein 3 like 1, *CREB3L2* CAMP responsive element binding protein 3 like 2, *DDIT3* DNA damage inducible transcript 3, *ERG* v-ets avian erythroblastosis virus E26 oncogene homologue, *ETV* ETS variant transcription factor, *EWSR1* EWS RNA-binding protein 1, *FGFR2* fibroblast growth factor receptor 2, *FLI1* Friend leukaemia integration 1 transcription factor, *FUS* fused in sarcoma, *GIST* gastrointestinal stromal tumour, *HCC* hepatocellular carcinoma, *HEY1* Hes-related family bHLH transcription factor with YRPW motif 1, *HMG2* high-mobility group protein 2, *KIT* v-kit Hardy-Zuckerman 4 feline sarcoma viral oncogene homologue, *MAML2* mastermind like transcriptional coactivator 2, *MYB* v-myb avian myeloblastosis viral oncogene homologue, *MYBL2* MYB protooncogene like 2, *NAB2* NGFI‑A binding protein 2, *NCOA2* nuclear receptor coactivator 2, *NR4A3* nuclear receptor subfamily 4 group a member 3, *NSCLC* nonsmall-cell lung cancer, *NTRK3* neurotrophic tyrosine receptor kinase type 3, *NUT* nuclear protein in testis, *NUTM1* nuclear protein in testis midline carcinoma family member 1, *PDGFB* platelet derived growth factor receptor beta, *PLAG1* pleomorphic adenoma gene 1, *POU5F1* POU domain, class 5, transcription factor 1, *PRKACA* protein kinase camp-activated catalytic subunit alpha, *PRKD1* protein kinase D1, *RCC* renal-cell carcinoma, *ROS1* ROS proto-oncogene 1, *SMARCB1* SWI/SNF related, matrix associated, actin dependent regulator of chromatin, subfamily b, member 1, *SS18(SYT)* synovial sarcoma translocation, chromosome 18, *STAT6* signal transducer and activator of transcription 6, *TFE3* transcription factor binding to IGHM enhancer 3, *TFEB* transcription factor EB, *TMPRSS2* transmembrane serine protease 2, *VHL* Von Hippel-Lindau, *WT1* Wilms tumor 1

Die aufgeführten Marker stellen eine persönliche Auswahl auf der Grundlage der kollektiven Expertenmeinung der Autoren dar

## Fazit für die Praxis

- Bei der initialen CUP-Situation („cancer of unknown primary“) leistet die Pathologie mit einem systematischen Ansatz, beginnend mit der histomorphologischen Auswertung, der schrittweisen Anwendung eines effektiven Panels von immunhistochemischen Markern und der präzisen Auswahl molekularer Analysen einen wesentlichen Beitrag zur Identifizierung der Primärtumoren.
- Es muss beachtet werden, dass immunhistochemische Marker gelegentlich aberrant exprimiert werden oder klon- und protokollspezifische Abweichungen aufweisen können; eine abschließende Diagnose sollte daher in Korrelation mit der Klinik- und insbesondere der Radiologie erfolgen.
- Beim echten CUP-Syndrom steht eine möglichst präzise phänotypische und molekulare Charakterisierung im Vordergrund mit dem vorrangigen Ziel, therapiesensitive Untergruppen des CUP-Syndroms zu identifizieren.
- Die Molekularpathologie ermöglicht neben der Unterstützung der Diagnostik im Idealfall auch eine Identifikation einer potenziell zielgerichteten Therapie.
- Bei zunehmender Verfügbarkeit von tumorspezifischen und individuellen Therapieschemata ist eine molekulare Aufarbeitung von besonderer Bedeutung.
